# Stage-specific neutrophil states in neonatal injury: from antimicrobial deficiency to pro-resolution repair

**DOI:** 10.3389/fimmu.2026.1884986

**Published:** 2026-08-14

**Authors:** Qiu-xiang Zhou, Zhi-qiang Bao

**Affiliations:** Department of Pediatrics, The First Hospital of Putian City, Putian, China

**Keywords:** bronchopulmonary dysplasia, context-dependent programs, hypoxic-ischemic encephalopathy, inflammation resolution, necrotizing enterocolitis, neonatal neutrophils, neonatal sepsis, neutrophil extracellular traps

## Abstract

Neonatal neutrophils are often described as immature effector cells with limited marrow reserves and reduced antimicrobial capacity. This account is clinically useful but incomplete: individual functions mature at different rates, neutrophil populations are heterogeneous, and observed phenotypes vary with gestational age, maternal health, tissue context, and time after injury. This Mini Review first places neonatal findings against canonical mature-neutrophil functions, then examines context-dependent programs in neonatal sepsis, hypoxic-ischemic encephalopathy, bronchopulmonary dysplasia, and necrotizing enterocolitis. We use state as an operational, cross-sectional description supported by phenotypic, functional, or molecular evidence, without assuming a stable lineage or a proven conversion from inflammatory to reparative cells. Temporal change may instead reflect emergency granulopoiesis, selective recruitment, survival, clearance, or population replacement. Human neonatal studies show that chemotaxis and extracellular-trap formation are often developmentally constrained, whereas phagocytosis, oxidative burst, granule content, and degranulation can be relatively preserved under specific assay conditions. Disease models further reveal competing roles for neutrophils in pathogen control, thromboinflammation, angiogenic support, and resolution. Conflicting findings on extracellular traps in necrotizing enterocolitis illustrate why model, microbial burden, intervention timing, and whether an experiment prevents trap formation or removes established traps must be distinguished. No clinically validated neonatal neutrophil-state classifier exists. Routine laboratory values provide clinical context and trajectories, whereas proposed interventions targeting extracellular traps, myeloperoxidase, trained immunity, lipid mediators, or efferocytosis remain experimental. Taken together, the evidence favors a context-dependent approach that preserves antimicrobial defense while testing ways to limit tissue injury.

## Introduction

Neonatal neutrophils are still often introduced as incomplete versions of adult neutrophils. Newborn infants have smaller storage pools, gestational-age-dependent kinetics, reduced complement support, altered chemotaxis, and age-appropriate activation thresholds (1–5). These features help explain rapid deterioration during infection and the clinical use of neutrophil counts and immature-to-total ratios.

A deficiency model is useful for recognizing limited reserve and impaired recruitment, particularly in preterm sepsis, but it cannot explain the full range of neonatal neutrophil biology. Neutrophils also influence immune regulation, macrophage efferocytosis, angiogenesis, lipid-mediator exchange, extracellular-vesicle signaling, and tissue homeostasis (6–9). Early life adds rapidly changing granulopoiesis, microbial exposure, and clearance capacity (3, 4, 10).

**Figure 1:**
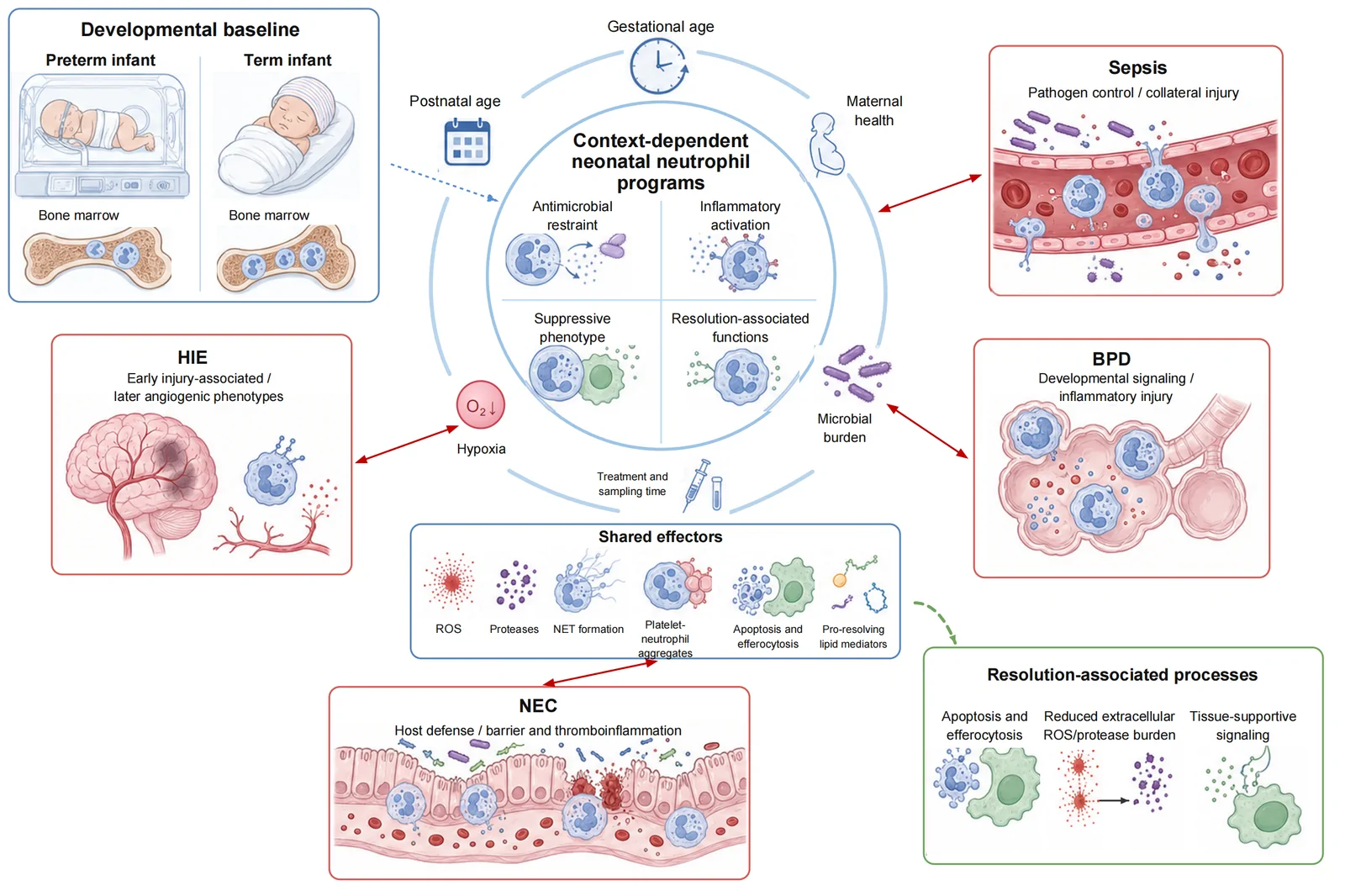
Context-dependent neonatal neutrophil programs across parallel disease settings. The schematic represents a network rather than a timeline. Developmental baselines in preterm and term infants and modifying factors—including gestational and postnatal age, maternal health, hypoxia, microbial burden, treatment, and sampling time—shape four potentially coexisting operational programs: antimicrobial restraint, inflammatory activation, suppressive phenotypes, and resolution-associated functions. Sepsis, HIE, BPD, and NEC represent parallel disease contexts. Shared effectors include ROS, proteases, NET formation, platelet–neutrophil aggregates, apoptosis and efferocytosis, and pro-resolving lipid mediators. Arrows denote contextual associations rather than proven lineage conversion or direct clinical causality; resolution-associated processes indicate reduced extracellular inflammatory burden and tissue-supportive signaling. BPD, bronchopulmonary dysplasia; HIE, hypoxic-ischemic encephalopathy; NEC, necrotizing enterocolitis; NET, neutrophil extracellular trap; ROS, reactive oxygen species.

This Mini Review examines sepsis, hypoxic-ischemic encephalopathy (HIE), bronchopulmonary dysplasia (BPD), and necrotizing enterocolitis (NEC) as parallel settings in which developmental baseline, tissue signals, and sampling time shape observable neutrophil programs (**Figure 1**). The framework does not assume that one disease or phenotype progresses into another.

## Literature-selection approach

This focused narrative review used PubMed/MEDLINE from database inception through 15 July 2026. Searches combined neonatal, newborn, preterm, or cord blood with neutrophil, granulopoiesis, chemotaxis, phagocytosis, degranulation, oxidative burst, extracellular traps, efferocytosis, sepsis, HIE, BPD, or NEC. We prioritized peer-reviewed human neonatal studies and primary neonatal models; organoid, ex vivo, adult, and non-neonatal studies were used when neonatal mechanistic evidence was unavailable. Reference lists of relevant articles were also screened.

## Canonical neutrophil effector functions and neonatal adaptation

Mature neutrophils move from blood to tissue through selectin-dependent rolling, integrin-mediated adhesion and transmigration, and chemotaxis toward microbial or damage signals. Complement and immunoglobulin opsonization support recognition and engulfment. Within phagosomes, NADPH oxidase-derived reactive oxygen species cooperate with granule enzymes to kill microbes; regulated degranulation also delivers antimicrobial proteins extracellularly. NET formation can immobilize pathogens but exposes histones, proteases, and MPO to surrounding tissue. Apoptosis followed by macrophage efferocytosis limits secondary release of these mediators and helps terminate inflammation (6, 7, 9).

These functions are not uniformly reduced at birth. Human cord-blood studies found phagocytosis and intracellular killing comparable with adult cells in some assays, preserved phagocytosis and oxidative burst against Candida in term and preterm samples, and robust stimulus-induced degranulation (11–13). Granule composition is selective rather than globally deficient: myeloperoxidase and defensins may be comparable, while bactericidal/permeability-increasing protein is lower (14). By contrast, impaired chemotaxis and reduced NET formation are among the more consistent developmental differences (5, 15). Gestational age, postnatal age, stimulus, opsonization, specimen handling, and assay endpoint therefore determine what is labeled impaired.

Phenotype also requires definition. Conventional blood neutrophils are commonly identified within CD45-positive myeloid cells by CD66b, CD15, and CD16, with CD10 and CD16 helping distinguish mature from immature populations; CD11b or CD64 upregulation and CD62L shedding can indicate activation. Density fractions, morphology, viability, and processing time should be reported because activated mature cells can overlap with immature or low-density populations. Markers do not establish function. In particular, a polymorphonuclear myeloid-derived suppressor cell (PMN-MDSC) designation requires demonstrated suppression of another immune-cell response rather than surface markers or transcriptomic resemblance alone (16, 17).

## Developmental baseline and maternal influences

At birth, neutrophil behavior reflects developmental programming rather than a single defect. Granulocyte-monocyte progenitors expand before birth, circulating neutrophils rise after delivery, and microbiota-dependent marrow granulopoiesis follows (1–4). Extremely preterm infants have the smallest reserve; very and late preterm infants remain vulnerable to inflammatory exposures even when counts approach term ranges (5, 18). Healthy newborns also show changing epigenetic regulation of the neutrophil-to-lymphocyte ratio during the first week (19).

Maternal health can shift this baseline. In a recent cohort, hypertensive disease with severe features was associated with lower initial neonatal neutrophil counts, although gestational age was the strongest predictor and most low counts resolved within 48 hours (20). Cord-blood neutrophils from pregnancies complicated by gestational diabetes showed impaired motility and post-phagocytic bactericidal activity in an ex vivo study (21). These associations should be interpreted with gestational age, fetal growth, labor, medication, and infection rather than as uniform maternal-disease phenotypes.

Resolution and immune memory add further variation. Early-life efferocytosis differs from adult clearance programs, while trained immunity can alter myeloid responses through metabolic and epigenetic reprogramming (10, 22). Sex should be reported with gestational and postnatal age because neonatal innate responses and preterm inflammasome or cytokine responses differ by sex in some cohorts and models (23, 24).

## Operational definitions and sources of apparent state change

Here, a neutrophil state is an evidence-linked snapshot defined by phenotype, function, or molecular profile; it is not a fixed lineage. Antimicrobial restraint denotes limited reserve, recruitment, phagocytosis, or oxidative burst. Inflammatory activation requires increased effector deployment, such as reactive oxygen species, degranulation, inflammasome output, NET formation, or platelet binding. Suppressive, angiogenic, or resolution-associated labels require corresponding functional or molecular evidence. Apoptosis, efferocytosis, and NET clearance are processes that shape these observations, not neutrophil states themselves.

Serial differences do not by themselves prove cell-intrinsic reprogramming. They may reflect maturation, emergency granulopoiesis, selective recruitment, altered survival, clearance, or replacement by newly generated neutrophils. We therefore use transition only when an experiment supports temporal continuity; otherwise we refer to time- and tissue-dependent phenotypes or population shifts.

Candidate mechanisms include efferocytosis-linked macrophage reprogramming, cytokine context, lipid-mediator switching, hypoxia and HIF-1α-dependent metabolism, and trained-immunity programs (9, 25–29). DEL-1 illustrates the context dependence: it can support efferocytic clearance in non-neonatal inflammation and IL-10-dependent emergency granulopoiesis in neonatal sepsis. HIF signaling can sustain survival and glycolysis yet also delay resolution when apoptosis and reverse migration are impaired. Single-cell and spatial methods can resolve coexisting populations and tissue localization, but lineage tracing or barcoding is needed to distinguish reprogramming from replacement (16, 30, 31).

Reproducible assignment therefore requires more than a label. Studies should report the sampled compartment, gestational and postnatal age, maternal and treatment context, cell-identification strategy, viability, maturation markers, and a prespecified functional endpoint. Serial sampling can show whether an observable program expands or contracts, while paired blood and tissue measurements can test whether circulating cells represent the injured compartment. Without these elements, terms such as activated, immature, suppressive, or reparative risk describing different populations across studies.

## Sepsis: antimicrobial insufficiency and inflammatory excess

Neonatal sepsis exposes the tension between antimicrobial insufficiency and inflammatory excess. Limited reserve and complement support are most consequential in preterm infants, yet increasing circulating cells has not consistently improved outcomes (32). In neonatal mouse polymicrobial sepsis, the IL-10/DEL-1 axis supported emergency granulopoiesis and survival, whereas IL-17A suppressed this pathway (26).

Inflammasome findings also depend on context. Ex vivo neonatal neutrophils showed reduced NLRP3 activation, caspase-1 activity, IL-1β release, and S100A8/A9 release after stimulation (33). Conversely, loss of myeloid KLF2 increased NLRP3 priming and mortality in neonatal mouse endotoxemia, and NLRP3 inhibition improved survival (34). These results describe different experimental systems, not a single low-to-high sequence.

NETs can immobilize microbes but can also promote platelet-neutrophil aggregates and collateral injury. NET inhibition or degradation reduced cytokines and mortality in a neonatal mouse infectious-peritonitis model (35), but this finding should not be generalized across pathogens or infants with limited neutrophil reserve. Pathogen burden, antimicrobial treatment, disease phase, circulating and tissue neutrophil abundance, and the consequences for microbial containment must be tested together. Serial human samples should also distinguish failure to form traps from impaired clearance of traps already present. BCG-induced trained immunity improved survival in experimental polymicrobial sepsis, but remains a model-specific preventive concept (36).

## Hypoxic-ischemic encephalopathy: time-dependent phenotypes

HIE is a sterile injury in which reactive oxygen species, cytokines, endothelial activation, and damage-associated molecular patterns engage innate pathways (37). Brain-border and peripheral leukocyte responses vary with developmental stage, barrier status, and concurrent infection (38).

Experimental studies first emphasized injury-associated effects. G-CSF can influence granulopoiesis, apoptosis, neurogenesis, angiogenesis, and inflammation (39). In newborn rats undergoing hypothermia after hypoxic-ischemic injury, citrullinated histone H3 and NLRP3-related signals were consistent with early NET formation (40). This does not show that NET-directed treatment improves human HIE.

In neonatal mice, infiltration was biphasic: day 1 neutrophils were hyperactivated and acute depletion reduced neural cell death, whereas day 7 Siglec-F-high neutrophils had an angiogenic phenotype and delayed depletion impaired vascular and oligodendrocyte regeneration (41). The signals driving this shift remain incompletely defined; candidates include the evolving DAMP and cytokine milieu, endothelial chemokine gradients, hypoxia/HIF-dependent metabolic programming, efferocytosis-linked macrophage reprogramming, and angiogenic repair cues. The two peaks could include both within-cell change and recruitment of distinct populations. Blood measurements alone cannot establish which explanation applies within brain tissue.

A separate neonatal mouse study linked LOX1-positive PMN-MDSC-like neutrophils, HIF-1α-dependent glycolysis, and lactate release to reduced microglial activation after acute hypoxia (17). We retain the PMN-MDSC label only where functional suppression was assessed; phenotype alone is described as PMN-MDSC-like. Human validation of these HIE phenotypes remains limited.

## Bronchopulmonary dysplasia: developmental signaling and inflammatory injury

During normal neonatal mouse lung development, neutrophil-derived 12-HETE supports alveolar-macrophage self-renewal and later maintenance (42). This physiological imprinting study does not establish 12-HETE deficiency or supplementation as a mechanism or treatment in human BPD; it shows why indiscriminate neutrophil suppression could disturb developmental signaling.

BPD arises in an immature lung exposed to oxygen toxicity, ventilation, infection, and antenatal or postnatal inflammation. The insult occurs during alveolar and vascular development, so the same neutrophil product may be antimicrobial in an airway and disruptive in a developing epithelial or endothelial niche. Persistent inflammation is associated with impaired alveolar and vascular development (43–45). In preclinical hyperoxia models, neutrophil infiltration, endoplasmic-reticulum stress, and later senescence accompany injury, and reversible MPO modulation has been protective experimentally (46).

Human preterm tracheal aspirates and neonatal mouse experiments link increased NET markers with BPD-like changes; in mice, heparin attenuated NET-associated disruption of WNT/β-catenin signaling (47). An inhaled lactobacilli-based biotherapeutic reduced neutrophil recruitment through the MMP-9/PGP pathway in an animal model (48). Single-cell neonatal hyperoxia studies identified sex-specific myeloid responses and MDSC-like neutrophil populations (49). The neutrophil-to-lymphocyte ratio has prognostic associations in infants but does not identify a specific neutrophil state (50).

## Necrotizing enterocolitis: host defense, barrier injury, and thromboinflammation

NEC combines immature epithelial defense, dysbiosis, ischemic stress, feeding exposure, and systemic inflammation. Human mucosal mass cytometry demonstrates local and circulating immune changes that include neutrophils (51), while chromatin alarmins provide a plausible link between epithelial injury and sustained activation (52). NEC organoids exposed to lipopolysaccharide showed increased TLR4 and apoptosis, and added neutrophils aggravated NEC-like injury (53). Organoids do not fully reproduce immune-cell interactions, stromal and vascular components, or host-microbiota crosstalk.

NET experiments have produced opposite results. In a formula/LPS/hypoxia neonatal mouse model, peptidylarginine deiminase inhibition reduced intestinal injury and mortality (54). In a dithizone/Klebsiella model, preventing NET formation increased bacteraemia, inflammation, and mortality (55). The contrast may reflect disease model, microbial burden, intervention timing, developmental age, neutrophil abundance, and whether an intervention blocks formation or degrades established traps. It precludes a general claim that NET inhibition is beneficial or harmful in NEC.

Human tissue and experimental work also implicates CD177-positive platelet-neutrophil aggregates in NET-associated thromboinflammation; disrupting aggregates or NETs, including with low-molecular-weight heparin, reduced disease severity in models (56). This identifies an experimental target, not a neonatal treatment recommendation, because anticoagulant exposure, intestinal bleeding, infection control, and intervention timing require separate neonatal evaluation. Preterm-pig studies further suggest post-NEC systemic suppression and impaired phagocytosis (57), while early-life antimicrobial peptides may influence defense and repair (58).

## Shared mechanisms and measurement limits

Across the four diseases, recurring mechanisms include NET-associated chromatin and protease exposure, context-dependent inflammasome activity, platelet-neutrophil interactions, and delayed clearance (35, 40, 47, 56, 59). Their effects vary with tissue oxygenation, microbial burden, vascular context, and the capacity of DNases and phagocytes to remove extracellular material.

NET-related measurements require particular caution. Preterm sepsis studies have measured cell-free DNA, nucleosomes, DNase activity, MPO, and elastase (60), but these readouts are neither specific for NET formation nor interchangeable. Stronger inference combines extracellular-DNA colocalization with neutrophil proteins and tissue imaging, supported by orthogonal assays such as citrullinated-histone-DNA or MPO-DNA complexes and appropriate controls. Even MPO-DNA ELISA and plasma H3Cit assays have important analytical limitations (61, 62). We use NET formation unless a cell-death mechanism was directly demonstrated; NETosis is reserved for such experiments.

Clearance is an active counterpart to activation. Failure to remove apoptotic neutrophils, extracellular chromatin, or proteases can prolong inflammation after the trigger declines, while efferocytosis and specialized pro-resolving mediators, including resolvins and protectins, can support macrophage reprogramming and immune silencing (7–9, 28). Most direct mechanistic evidence for these pathways is non-neonatal, so neonatal timing, safety, and tissue effects remain to be established.

## Current care and experimental therapeutic directions

Current neonatal care is not based on a neutrophil-state classifier. Sepsis management relies on prompt cultures, antimicrobial treatment, source control where relevant, and organ support; neutropenia prompts evaluation of gestational age, maternal disease, infection, medication, and immune causes. Prophylactic GM-CSF increased neutrophil counts in extremely preterm, small-for-gestational-age infants but did not reduce sepsis or improve survival in the PROGRAMS trial (63). Growth-factor treatment is therefore not routine state-directed therapy.

Established disease care acts upstream or downstream of neutrophils rather than on a defined neutrophil program. Therapeutic hypothermia improves outcomes in eligible neonatal HIE (64). Lung-protective ventilation, careful oxygen targeting, nutrition, and selected postnatal corticosteroid use support infants at risk of BPD; the DART trial showed that low-dose dexamethasone facilitated extubation in chronically ventilated infants, not that it corrected a neutrophil state (65). NEC care remains bowel rest, decompression, antibiotics, physiological support, and surgery when indicated.

No clinically validated neonatal neutrophil-state classifier currently exists. Serial ANC, immature-to-total ratio, neutrophil-to-lymphocyte ratio, platelets, CRP, procalcitonin, cultures, and organ-support requirements are clinical context or trajectory markers, not state-specific biomarkers. A practical research design can begin with serial samples already obtained in intensive care, then add prespecified functional and tissue-localized assays in well-phenotyped cohorts. NET inhibition, MPO modulation, heparin for thromboinflammation, trained-immunity strategies, lipid mediators, and efferocytosis-targeted approaches remain experimental. Their evaluation should combine developmental age, disease phase, pathogen control, tissue-localized evidence, and safety endpoints rather than total neutrophil abundance alone (**Table 1**).

**Table 1:** Evidence hierarchy and translational interpretation of neonatal neutrophil phenotypes.

Clinical context and timing	Operational phenotype or function	Evidence hierarchy and human-neonatal validation	Clinical context/trajectory markers and mechanistic assays
Sepsis: early infection and post-control inflammation	Limited reserve or recruitment; stimulus-dependent inflammasome output; NET- and platelet-associated injury. Requires counts plus functional or tissue evidence (26, 32, 33, 35).	Human preterm cohorts and ex vivo neonatal blood; neonatal mouse polymicrobial sepsis, endotoxemia, and peritonitis. Human validation is moderate for count trajectories and limited for proposed functional states.	Context/trajectory only: ANC, immature-to-total ratio, platelets, CRP/procalcitonin, cultures, organ support. Mechanistic: CD64/CD11b, inflammasome assays, colocalized NET imaging, CitH3-DNA or MPO-DNA with controls. None is a validated state classifier (60).
HIE: acute injury and later tissue response	Early hyperactivated/infiltrating phenotype versus later Siglec-F-high angiogenic phenotype; LOX1-positive suppressive phenotype only when functional suppression is shown (17, 40, 41).	Neonatal rat hypothermia and neonatal mouse depletion/metabolic studies. Direct human neonatal validation of these phenotypes is currently absent.	Context/trajectory only: encephalopathy grade, hypothermia timing, MRI, serial blood counts. Mechanistic: tissue localization, NET imaging, Siglec-F/angiogenic profile, glycolysis/lactate, functional suppression assays.
BPD: immature lung exposed to oxygen, ventilation, and infection	Persistent recruitment, MPO stress, and NET-associated injury; developmental 12-HETE signaling is a separate physiological function (42, 46, 47).	Human preterm tracheal aspirates and observational blood cohorts; neonatal mouse hyperoxia and biotherapeutic models. Human validation is partial for association, not state-directed treatment.	Context/trajectory only: oxygen/ventilation exposure, infection history, ANC, neutrophil-to-lymphocyte ratio. Mechanistic: airway NET colocalization, MPO activity, lipid mediators, single-cell profiles. NLR is not state-specific.
NEC: epithelial injury, infection, thrombosis, and post-injury suppression	Mucosal recruitment; model-dependent NET-mediated containment or injury; CD177-positive platelet-neutrophil aggregates; later impaired phagocytosis (51, 54–57).	Human neonatal mucosa and blood, human-derived organoids, two contrasting neonatal mouse models, and preterm pigs. Human validation is strongest for association; therapeutic causality remains preclinical.	Context/trajectory only: gestational/feeding context, ANC, platelets, inflammatory and coagulation trends, clinical stage. Mechanistic: tissue NET imaging, aggregates, organoid assays, phagocytosis. NET assays are not interchangeable.
Cross-cutting resolution-associated processes	Neutrophil apoptosis, efferocytosis, reduced extracellular chromatin/protease burden, macrophage reprogramming, and lipid-mediator switching (7–9, 28).	Predominantly adult/non-neonatal mechanistic systems, with limited neonatal animal and observational support. Human neonatal validation is low.	Context/trajectory only: falling inflammatory markers and organ-support needs after infection control. Mechanistic: efferocytosis assays, spatial imaging, DNase activity with NET-specific evidence, lipid-mediator profiling.

## Discussion

Neonatal neutrophil biology is better represented by context-dependent programs than by a single maturation deficit or a fixed sequence of states. A preterm infant may encounter antenatal inflammation, antibiotics, oxygen exposure, feeding transitions, infection, bowel injury, and chronic lung disease within weeks; each exposure can alter production, recruitment, survival, and clearance.

Translation remains limited by evidence source. Human studies usually sample peripheral blood, whereas decisive events occur in brain borders, airway fluid, alveoli, intestinal mucosa, or microvasculature. Animal models permit timed depletion and tissue sampling, but postnatal age and organ maturation do not map directly onto human gestation. Organoids isolate selected epithelial interactions, and adult studies supply mechanisms without establishing neonatal effects. Multicenter sampling with harmonized pre-analytical handling is needed because delayed processing can alter activation markers, density, viability, and NET readouts. Future work should pair surface phenotype with function, spatial location, sex, gestational age, maternal context, and time after injury.

The practical aim is resolution-preserving immunomodulation: maintain microbial killing when reserve is limited, while testing whether excessive chromatin, protease exposure, platelet coupling, or failed efferocytosis can be reduced without impairing host defense or development. Because antimicrobial, inflammatory, suppressive, and resolution-associated programs may coexist in the same infant, a single cross-sectional value is unlikely to guide treatment. This formulation retains the clinical value of the deficiency model and sets a higher evidentiary threshold for claims of reparative phenotypes or therapeutic state transitions.
